# Comparative ruralism and ‘opening new windows’ on gentrification

**DOI:** 10.1177/2043820617752035

**Published:** 2018-02-26

**Authors:** Martin Phillips, Darren P Smith

**Affiliations:** University of Leicester, UK; Loughborough University, UK

**Keywords:** comparative ruralism, displacement, rural gentrification, rurality, scale, urbanity

## Abstract

In response to the five commentaries on our paper ‘Comparative approaches to gentrification: lessons from the rural’, we open up more ‘windows’ on rural gentrification and its urban counterpart. First, we highlight the issues of metrocentricity and urbanormativity within gentrification studies, highlighting their employment by our commentators. Second, we consider the issue of displacement and its operation within rural space, as well as gentrification as a coping strategy for neoliberal existence and connections to more-than-human natures. Finally, we consider questions of scale, highlighting the need to avoid naturalistic conceptions of scale and arguing that attention could be paid to the role of material practices, symbolizations and lived experiences in producing scaled geographies of rural and urban gentrification.

## Introduction

Our article aimed to stimulate discussion of how framings other than the urban might contribute to comparative studies of gentrification, as well as considering the impact of comparative research on conceptualizations of gentrification. Our interlocutors provide positive commentary, noting we have opened a ‘window into a wider (than English speaking) world’ (Bernt, 2018: 2) as well as making critical points. Here, we respond to these latter comments, through clarifying our original arguments and using the comments to develop some further arguments, which we have structured around three themes.

## Rurality and urbanity

The two urban scholars raise questions about the significance of our paper for studies of urban gentrification, suggesting that we failed to produce ‘much lessons’ for this research (Bernt, 2018: 3), and questioning whether conceptualizations of rural gentrification ‘nurture important ongoing debates related to the expansion of cities and urbanization processes’ (López-Morales, 2018: 4). Bernt further argues that we ‘play it too safe’ and fail to ‘make use of the ammunition collected’ to ‘directly name’ what we view as ‘false projections and misled conceptualisations’. While rather sceptical that such language creates the ‘fresh atmosphere’ that Bernt desires, we begin this response by reiterating that a key argument of our article was that gentrification studies have tended to employ a ‘metropolitan centricity’, not only through privileging a narrow range of cities in the Global North but also through setting up the urban as a privileged viewpoint through which to interpret the world.

Thomas et al.’s (2011) concept of ‘urbanormativity’ is useful in elaborating this argument, as it refers to the practice of taking urban space, or, particular subsets of it, as constituting the ‘normal’ space of operation of social processes, while alternative locations are ‘othered’ as spaces of absence or abnormality. Such arguments can be applied to gentrification studies, where a range of ‘other geographies of gentrification’ (Phillips, 2004) have been identified. Moreover, we would argue that the commentaries of Bernt, López-Morales and Nelson provide enactments of metrocentricity/urbanormativity. Bernt, for example, appears to identify New Age professionals as an urban phenomenon, an argument that neglects the significance of rurality in many forms of ‘new-ageism’ (e.g. Chevalier, 1981; Halfacree, 1995). More generally, Bernt (2018: 4) suggests that rural changes should be conceptualized as ‘part of the re-configuration of the urban’, a viewpoint that gives no scope for agency to emerge from within rural areas. Nelson also seems to view rural gentrification as a process originating in the urban, albeit one that has moved down the urban hierarchy into rural locations in countries such as the United Kingdom and United States, a diffusionist claim we explicitly contested in our article, both empirically for its neglect of sites of rural gentrification dated soon after coinage of the term by Glass (1964) and more theoretically through the concept of genetic comparisons.

López-Morales develops very similar claims to Bernt and Nelson, drawing explicitly on the notion of planetary urbanization that has informed his work (Lees et al., 2016; López-Morales et al., 2016), to suggest that rural/urban divisions may not make sense ‘in a world where everything is essentially urban’ (López-Morales, 2018: 4). Both López-Morales and ourselves make reference to the work of Brenner and Schmidt, although we do as an illustration of how the urban is often elevated to a privileged position. While there is value in their work, we agree with Walker’s (2015: 188) argument that planetary urbanization ‘effectively erase the rural’ via constructing spaces that may be identified as ‘rural’ as completely entwined with the dynamics of urbanization, a perspective which he claims is problematic as ‘the rural “Other” has not been fully internalized by the urban’ (see also Roy, 2016).

Walker adds that recognizing rural otherness does not imply such spaces are disconnected from the urban, but rather that the presence of relational connections does not reduce localities to urbanity. Such arguments have a long history in rural studies, where there have not only been repeated calls to recognize encompassing relations with urban spaces (e.g. Cloke, 1989; Hoggart, 1990) but also recurrent arguments about the need to recognize the specificities of rural spaces and places. Here, connections can be made to Hines’ (2018) favouring of particularist ethnographies, although his desire to identify recurrent stories invokes universalistic or generic elements. Reference might also have been made to calls for multisite or global ethnographies (Marcus 1995; Burawoy 2001), which enact relational comparisons. Comparative strategies, hence, cannot be equated to the adoption of particular methodologies.

López-Morales’ (2018: 4) comment that our paper exhibited a ‘lack of sensitiveness regarding the challenging different geographies and analytical categories a properly comparative reflection should present’ also raises questions concerning the conduct of comparative research. His assessment seems to be based on a privileging of North–South comparisons over all other comparisons. We were therefore heartened by Bernt’s (2018: 2) assessment that our paper ‘productively demonstrate that a North–South binary which has become customary in much of the urban studies world is neither necessary, nor sufficient for making comparison work as a strategy’. This is not to say that North–South comparisons are unimportant, with rural scholars asking many of the same questions that López-Morales raises concerning the applicability of conceptions of space and transformation produced in the Global North to the Global South (e.g. Korf and Oughton 2006; Murray 2008). We would argue that there is a need for a ‘comparative ruralism’ that, like its urban counterpart, extends its focus across the Global North and South, although would also point to the significance of other forms of spatial differentiation. López-Morales himself has raised the issue of regional differentiation in his discussions of ‘Latin American gentrification’ (López-Morales et al., 2016) and the ‘Global East’ (Shin et al., 2016). There is research on rural gentrification in each of these contexts (e.g. Jokisch, 2002; Qian et al., 2013), and we ourselves are exploring gentrification in the context of the Global East (Phillips, 2016; Smith, 2017). Having said this, we would suggest that there is value and challenge in comparisons even within the Global North, not least in relation to the issue of language raised by Gkartzios (2013) and discussed by Halfacree (2018).

Halfacree’s commentary also provides an illustration of how arguments over rural specificity may be enacted within gentrification studies, remarking that a simple appropriation of gentrification from urban studies might be viewed as a ‘colonizing’ attitude towards the rural. As we noted in our article, many studies of rural gentrification include cautionary remarks about direct transfer of the concept of gentrification from urban to rural contexts. We suspect that many rural scholars refrain from employing the concept because of its perceived connections to urban space.

Halfacree also argued that rural population change needed to have been mapped before researchers began to engage with the concept of rural gentrification. This is an interesting claim, particularly given Nelson et al.’s (2010) view that there is ‘scant understanding of the extent of rural gentrification at a more macroscale’. We would, however, argue that mapping is itself conditioned conceptually, and, hence, by the associated circulatory sociologies of translation. We would also point to references to rural gentrification prior to the study of Phillips (1993) (e.g. Cloke, 1979; Parsons, 1980; Fitchen, 1991) as well as the ‘feet on the ground’ recognition of ‘class-based “up-scaling”’ made without reference to gentrification outlined by Halfacree (2018: 2). For us, it is important to not only recognize the presence of rural gentrification where the term is not employed but also consider reasons as to why the term was not employed.

## Rural gentrification and questions of displacement

Halfacree provides arguments as to why the term might have been resisted by rural researchers, suggesting that reservations might stem from uncertainties relating to the presence of displacement as well as the concept’s perceived connections to urban space discussed above, and the postmodern and cultural turns discussed in our paper. Specifically, Halfacree argues that working class displacement preceded the arrival of middle class residents, and hence these were not causally related as implied in gentrification. These are important issues to consider and Halfacree may well be right to signal these as possible barriers to the incorporation of gentrification into rural studies.

Displacement has, indeed, been a facet of gentrification that has been rather neglected within rural studies, despite claims as to its critical significance to the overall concept of gentrification (Slater, 2006, 2008). Having said this, it is important to note that the points Halfacree makes could be seen to have relevance to both urban and rural space and, hence, arguably do little to explain differences between urban and rural scholars. Many accounts of urban gentrification, for instance, have pointed to the abandonment of areas prior to gentrification, with Marcuse (1985) providing an insightful discussion of the extent of abandonment in New York and its relationship to gentrification and displacement. In line with Halfacree’s (2018: 4) remarks about the need to situate discussions of demographic change within a wider framing of ‘ongoing renewal of class polarisation within society as a whole’, Marcuse presents abandonment and gentrification as distinct manifestations of class restructuring, whereby there was a decline in working class employment opportunities and associated effective demand for housing, which led to property abandonment in some neighbourhoods, alongside growth of professional service employment that led to demands for office development and gentrified properties. While Halfacree points to the presence of a different, and in many instances, earlier phase of restructuring linked to reductions in agricultural work, Marcuse highlights how gentrification may occur alongside abandonment and, indeed, may involve replacement in areas that have been abandoned. As discussed in Phillips (2005), abandonment can be seen as one way that spaces are ‘made ready for gentrification’, not least because it indicates the devalorization of properties that can constitute the emergence of a rent gap that comes to be closed through gentrification.

Marcuse also discusses how displacement may occur in a range of forms. We feel this discussion might be of value to rural studies, pointing to how displacement may operate both over time and in far from direct ways. Marcuse, for instance, notes how residents can be displaced quite immediately and directly, through combinations of physical force, material neglect and economic pricing – or what he identifies as ‘direct last-resident displacement’ (Marcuse, 1985: 206). Work on the cessation of tied housing (e.g. Newby, 1977; Bowler and Lewis, 1987) as well as the outbidding of ‘local people’ in housing markets (e.g. Shucksmith, 1991; Liu and Roberts, 2013) might be viewed as illustrative of the presence of this form of displacement. Marcuse adds, however, that displacement can occur across a series of residents, with several residents leaving prior to the departure of the final non-gentrifier resident. Such a concept has not, to our knowledge, been applied to rural gentrification but would seem to be of potential relevance. Cloke et al. (1995), for instance, make reference to people accessing rural areas through ‘marginalised channels of entry’ including staying with family and friends (see also Cloke et al., 2000), and many of these ‘rural residents’ appear likely to move away from these places of residence and hence could be viewed as displaced. There is also clear evidence that some offspring of contemporary rural residents are unable to access housing in these localities and move away in search of affordable homes. Hence, while in many villages, there may currently be a continuing ‘working-class’ presence, this may not continue into the future. This connects to a third form of displacement identified by Marcuse (1985: 206), namely, ‘exclusionary displacement’, focused on how the gentrification of properties prevents replacement of non-gentrifier households by other non-gentrifier households. There may even in some instances not be any direct displacement, but there is exclusion of certain forms of in-migration, a description that might be applicable not only to the ‘re-filled emptied’ rural space of Halfacree’s commentary but also to the brownfield sites of urban new-build gentrification, where authors such as Davidson and Lees (2005, 2010) have explicitly employed Marcuse’s notion of exclusionary gentrification as well as his fourth form of displacement, ‘displacement pressure’. This relates to the changing character of places and how this can result in a location becoming ‘less and less liveable’ (Marcuse, 1985: 206) for a group of existing residents. This declining liveability may be quite material in form, such as, for instance, when the loss of bus services prevents rural residents accessing work, retail or welfare services. It also takes more experiential and affective dimensions, whereby people come to feel that they do not belong to a place.

We feel that a series of ‘new windows’ between urban and rural studies could be opened-up through these notions of displacement. The notion of ‘displacement pressure’ quite directly connects to the ethnographic perspective outlined by Hines (2018), which, in turn, exhibits parallels to Halfacree’s (2018: 4) call for gentrification to be viewed as a ‘coping strategy’ to deal with contemporary ‘neo-liberal existence’, albeit conjoined with recognition of their impacts on other people, a call that has strong resonances with the work of Schlichtman et al. (2017). The concept of displacement pressure also highlights how displacement does not necessarily involve movement of people, an argument that could be considered in relation to the literature on both ‘new-build’ and ‘green’/‘ecological’/‘low-carbon’ gentrification. The latter forms of gentrification are mentioned by Bernt in relation to elaborating an argument about a blurring of rural and urban spatial distinctions, although his argument in our view reduces rurality to a space of nature. While we have both argued that notions of nature and the natural may be important constituents of symbolizations and senses of rurality (Phillips et al., 2008; Smith and Phillips, 2001), both nature and rurality are complexly and variously constructed and apprehended (Phillips, 2014). Just as we have argued that attention could usefully be paid to the significance of different rural landscapes in processes of gentrification (Phillips and Smith, 2018: 20; see also Smith et al., 2018), so we would argue that the presence and differences in ‘more-than-human’ natures or environments need to be recognized. The restored natures of formerly polluted ‘brownfield’ sites exhibit differences, as well as similarities, with the natures of public parks, intensive agriculture and wilderness, and these are probably poorly served by dualistic constructions of rurality and urbanity. Furthermore, as argued in Phillips (2010b: 51), if arguments over displacement are extended to include the more-than-human, even new-build developments of land not previously used for residential use could be viewed as creating displacement, of both direct and in-direct kinds, in that spaces are rarely uninhabited by ‘plants, animals and other forms of biodiversity’. Such spaces of nature, as well as the environments of buildings, are often the focus of experiential and affective attachments and hence can be the foci of ‘displacement pressure’ linked to the transformation of place, or as Davidson (2009: 227) puts it, drawing on the work of Heidegger and Lefebvre, the ‘transformation of lived space’ (see also Phillips, 2002).

## Scaled geographies of gentrification

Nelson (2018: 2) argues that a ‘sensitivity to scale can expand our understandings of gentrification’. Scale is, however, a highly contested concept, with calls to both do away with the concept (Marston et al., 2005) and reconceptualize it (Smith, 1992; Howitt, 1998; Marston, 2000). In both cases, criticisms are made about scale being ‘naturalized’ as a conceptual pregiven. We were, therefore, surprised that Nelson’s commentary contained little reflection on the meaning and construction of scale, as well as being uncertain about some claims made about the employment of scale within gentrification studies. For instance, there seems to be a suggestion that Zukin (1989) and Ley (1996) both conduct their analysis at the interurban scale of ‘a system of cities’ (Nelson, 2018: 3), although we would apply this phrase only to the latter study. We would also question Nelson’s illustration of how scale can inform understandings of the ‘geography of the concept of rural gentrification’, which implies that the presence of studies of gentrification within London acted to produce ‘heightened awareness’ of gentrification in the surrounding urban and rural areas, while in the United States scholars preferred to make use of concepts such as ‘“counterurbanization” or “amenity migration”’ (Nelson, 2018: 4–5). However, not only have conceptions of counterurbanization been more widely adopted in the United Kingdom than rural gentrification (Phillips, 2010a), but early references to rural gentrification emerged in areas of the United Kingdom quite distant to London, such as Devon and Warwickshire (Cloke, 1979), Nottinghamshire (Parsons, 1980), and Gower, Wales (Phillips, 1993). Likewise, the claim that studies of rural gentrification in the United States had to travel greater distances from their urban centres might be questioned both through reference to the work of Fitchen (1991) in New York state and Frieberger (1996) in Texas, and by asking why the concept of gentrification seemingly travelled so strongly to spaces remote from urban centres without being applied to spaces more proximate to urban areas. More generally, we would caution against too direct a reading of the geography of rural gentrification from the geography of rural gentrification scholarship.

There also seemed in Nelson’s commentary to be a sense that scale is constitutive of different senses of rurality. This is a further interesting new ‘window’ through which researchers might usefully peer. Having said this, we are mindful of Brenner’s (2001) comments about the unreflective conflation of conceptions of scale with concepts such as space and place, and would argue that scale is not strongly constitutive of all senses of rurality, and that in considering its significance, attention needs to be paid as to how scale is being viewed.

Nelson’s discussion of scale is, we would suggest, very Cartesian in tone, making repeated references to measures of travel times and distances, as well as settlement size. Such a sense of scale is consistent with what Lefebvre (1991) has described as ‘abstract space’, which Dimendberg (1998: 24) argues can be viewed as a representation of space in which material spatial practices and lived spaces of representation ‘recede in importance’. Much of the critique of naturalized conceptions of scale has focused on detailing the material practices and relations through which scales come to be socially constructed, and it may be that Nelson’s discussion of production-side and demand-side interpretations of gentrification could connect to such perspectives. Personally, we find the differentiation of interpretations into such sides overly dualistic and also do not consider that particular forms of comparison are tied to particular scales of analysis as Nelson seems to imply. There are, however, scalar dimensions to the remarks made in our paper about metrics within comparative studies that we should have highlighted: many instances of gentrification may go unrecognized because indicative material is obscured within data sets constructed at a greater spatial scale than gentrification is being realized at within a locality.

We would also argue that in any scaling of gentrification studies, attention needs to be paid to the presence of a range of scalar representations, including ones that that take less abstracted forms, and also to lived experiences of scale. We have recently made use of indices of low-population density and low-connectivity to urban centres of employment to identify areas of wilderness gentrification (Smith et al., 2018), stressing that while these indices connect to popular representations of wilderness as places devoid both of the presence of and connections to centres of large scale human settlement, these locations are symbolized in a range of ways and people can find wilderness experiences in areas relatively proximate in abstract space. Howitt’s (2002) argument that consideration of scale needs to connect to studies of lived experiences would seem to be of clear significance here, with a further window of opportunity being to inject scale into discussions of lived space and representations of space that have emerged in gentrification studies (e.g Phillips, 2002; Davidson, 2009; Davidson and Lees, 2010).

## Concluding comments

We have sought to open up more windows on rural gentrification to bring, to borrow Bernt’s phraseology, some ‘fresh air’ into this subject of study, and its urban counterpart. Our first window highlighted issues of metrocentricity and urbanormativity within gentrification studies, as well as discussing the spatial framings of comparative study and arguing for the development of a comparative ruralism. Through the second window, we examined displacement and its operation within rural space as well as considered gentrification as a coping strategy for neoliberal existence and gentrification and more-than-human natures. The final window related to questions of scale, highlighting debates about the significance and meaning of the concept of scale, as well as considering the role of scale in the formation of rurality and gentrification. We thank our interlocutors for their comments and stimulating us to think about further areas of exploration.
